# Pectin-Tannic Acid Nano-Complexes Promote the Delivery and Bioactivity of Drugs in Pancreatic Cancer Cells

**DOI:** 10.3390/pharmaceutics12030285

**Published:** 2020-03-22

**Authors:** Sumeet S. Chauhan, Advait B. Shetty, Elham Hatami, Pallabita Chowdhury, Murali M. Yallapu

**Affiliations:** 1 Department of Immunology and Microbiology, School of Medicine, University of Texas Rio Grande Valley, McAllen, TX 78504, USA; sumeet.chauhan01@utrgv.edu; 2 Department of Pharmaceutical Sciences, University of Tennessee Health Science Center, Memphis, TN 38163, USA; ashetty@uthsc.edu (A.B.S.); ehatami@uthsc.edu (E.H.); pchowdhu@uthsc.edu (P.C.); 3 South Texas Center of Excellence in Cancer Research, School of Medicine, University of Texas Rio Grande Valley, McAllen, TX 78504, USA

**Keywords:** pancreatic cancer, nanoparticles, gemcitabine, irinotecan, 5-fluorouracil, chemotherapy, anticancer drugs

## Abstract

Pancreatic cancer (PanCa) is a lethal disease. Conventional chemotherapies for PanCa offer severe systemic toxicities. Thus, the development of a successful nanomedicine-based therapeutic regimen with augmented therapeutic efficacy is highly sought. Naturally occurring pectin and modified pectin-based drug delivery systems exhibit remarkable self-targeting ability via galactose residues to various cancer cells. Herein, we developed and used an innovative approach of highly stable nanocomplexes based on modified pectin and tannic acid (MPT-NCs). The nanocomplex formation was enabled by strong intermolecular interactions between pectin and tannic acid under very mild conditions. These nanocomplexes were characterized by particle size and morphology (DLS, TEM, and SEM), FT-IR spectroscopy, and zeta potential measurements. Additionally, MPT-NCs were capable of encapsulating anticancer drugs (5-fluorouracil, gemcitabine, and irinotecan) through tannic acid binding. The in vitro bioactivity of these drug MPT-NCs were evaluated in pancreatic cancer adenocarcinoma (PDAC) cell lines (HPAF-II and PANC-1). A dose-dependent internalization of nanocomplexes was evident from microscopy and flow cytometry analysis. Both proliferation and colony formation assays indicated the anticancer potential of pectin drug nanocomplexes against PDAC cells compared to that of free drug treatments. Together, the pectin-based nanocomplexes could be a reliable and efficient drug delivery strategy for cancer therapy.

## 1. Introduction

According to the American Cancer Society, approximately 57,600 people will be diagnosed with pancreatic cancer (PanCa) and about 47,050 people will die of this disease in the United States in 2020 alone [1]. PanCa is the fourth leading cause of cancer-related deaths in the United States. The five-year survival rate for PanCa is 7%, making it one of the lethal forms of cancer [2]. It is typically diagnosed at a later stage, so surgery becomes difficult to perform, and chemotherapy is a suitable option. 5-Flurouracil (5-FU), gemcitabine (GEM), and irinotecan (IRI) are some of the FDA approved mono or combination chemotherapeutics for PanCa [3,4,5]. However, conventional chemotherapies are often associated with numerous systemic side effects and lead to drug resistance, which results in low therapeutic benefit(s). Therefore, delivery of therapeutics using a suitable carrier would be more efficient than conventional counterparts [6]. The immediate requirement is to have a suitable packaging carrier for clinical progression that needs to be biodegradable and biocompatible with a low immunogenic profile. A significant amount of research exists in the design and development of various types of nanocarriers [4,6,7] for accelerated clinical applications.

Nanotechnology often overcomes the chemotherapy associated issues [8]. Nanotechnology- based carriers primarily aimed to improve the therapeutic efficacy of anticancer agents by increasing bioavailability, solubility, and retention time at the tumor sites. On the other hand, nanoparticle technology offers the improved tumor targeting capability of therapeutic drug(s), which in turn reduces the systemic side effects. Such events reduce dosing frequency in a chemotherapy regimen. Among various types of nanocarriers, naturally occurring compound-based nanocomplexes have been receiving extensive attention in drug delivery applications [9,10,11]. These molecules can produce a spontaneous self-assembly or complexation with therapeutic agents. Polysaccharides such as chitosan, alginate, and pectin are among a few of the studied nanocarriers capable of forming self-assemblies with a wide range of molecules and therapeutic agents [12,13]. Pectin is a highly suitable molecule for drug delivery applications [14,15,16,17] due to its superior water solubility. Pectin is a naturally occurring polysaccharide commonly obtained from the peel of citrus or apple fruit. This is a commonly used thickening or stabilization agent in the food industry [18,19,20,21]. It is believed that the soluble fibers of apples and citrus that are commonly made into jams, preserves, and dietary supplements can improve the digestive associated health and minimize some gastrointestinal and metabolic disorders [18,22,23,24]. Additionally, pectin is a pharmaceutic ingredient to produce gel or gel capsules due to its minimal toxicity and low cost [20,25]. Pectin-based formulations are suitable for oral dosage forms for colon targeting where content can be released by the degradation of pectin with microbial enzymes [26]. Pectin is rich in β-galactose units, which can bind specifically to the galectin-3 adhesive molecule, which is commonly overexpressed in various types of cancers [15,26]. Thus, a pectin-based nanocarrier is a suitable for therapeutic delivery.

However, it is highly difficult to attain a stable colloidal nanosystem using pectin. This may be attributed to robust intra- and inter-particle aggregation and adhesion behavior. Additionally, pectin(s) are hydrophilic in nature and often associated with pre-mature release of therapeutics. Such property makes the pectin nanocomplexes highly challenging to scale-up, reproduce, and redisperse for efficient drug delivery purposes. Ca^2+^ or Zn^2+^ are commonly used cross-lining agents to produce pectin macro-/micro-gel particles [27,28,29]. The opposite divalent ions cross-link with pectin polymer chain(s) following an egg box mechanism [30,31]. Nevertheless, such cross-linking is not highly favored in therapeutic delivery because therapeutic content may be leached out before reaching the intended target(s). Considering these aspects, we introduce a simple cross-linking step to achieve stable nanocomplexes with enhanced preparative aspects and drug entrapment, for the improved bioactivity of drugs. Tannic acid (TA) is a polyphenolic compound obtained from plant origin. Previous studies have shown multiple human health benefits of TA including the anti-cancer ability [32,33]. The hydroxyl bond in tannic acid allows for hydrogen bonding with the drug molecule, in turn increasing its water solubility [34]. TA has been widely used as a pharmaceutical excipient in formulations of various drugs, peptides, vaccines, etc. Incorporation of TA in the formulation is known to enhance bioavailability and increase entrapment of the cargo.

Therefore, the goal of this study was to investigate the potential utility of modified pectin and tannic acid cross-linked nanocomplexes (MPT-NCs) for cancer therapeutic application. The effect of tannic acid content in modified pectin formulation dictates the formulation characteristics including particle size and stability. This study focusses on the particle size, surface charge, and stability of the nanocomplex drug delivery system. Optimized pectin-tannic acid nanocomplexes were further employed to deliver therapeutic agents (GEM, 5-FU, and IRI) for improved therapeutic activity in pancreatic cancer cells. Due to the multi-aspect approach of the nanocomplex, it could selectively target tumor cells for a better treatment of pancreatic cancer.

## 2. Materials and Methods

### 2.1. Chemicals

Tannic acid (TA, Catalogue No. 403040) was purchased from Sigma Aldrich (St. Louis, MO, USA). Pectin A (pectin from apple), pectin C (pectin from citrus), and modified pectin (MP, PectaSol-C, modified citrus pectin) were purchased from ecoNugenics (Santa Rosa, CA, USA). All other laboratory reagents, chemicals, and cell culture plastic were purchased from Fisher Scientific (Pittsburgh, PA, USA) or Sarstedt, Inc. (Newton, NC, USA).

### 2.2. Cell Culture

Human pancreatic cancer cell lines, HPAF-II (HPAF-II is a human pancreatic adenocarcinoma cell line derived from peritoneal ascitic fluid of a 44 year old Caucasian male with primary pancreatic adenocarcinoma and metastases to the liver, diaphragm and lymph nodes) and PANC-1 (a human pancreatic cancer cell line isolated from a pancreatic carcinoma of ductal cell origin of 56 years Caucasian male) were purchased from the American Type Culture Collection (ATCC, Manassas, VA, USA) and cultured using cell culture media, Dulbecco’s Modified Eagle Medium-Dulbecco’s Modified Eagle Medium:Nutrient Mixture F-12 (DMEM/F12) and DMEM (Cyclone Laboratories, Inc., South Logan, UT, USA), respectively. The media were supplemented with 10% fetal bovine serum (Atlanta Biologicals, Flowery Branch, GA, USA) and 1% (*w/v*) penicillin-streptomycin (Gibco, Thermo Fisher Scientific, Grand Island, NY, USA). These cells were maintained at 37 °C in a humidified atmosphere (5% CO_2_ and 95% O_2_ atmosphere). Cells were grown in T-75 flasks and, once they reached 70–80% confluency, trypsinized, and seeded for cellular uptake, cell viability, and colony formation experiments.

### 2.3. Pectin-Tannic Acid Nanocomplex Formation

Nanocomplexes of pectin (pectin A, pectin C, and modified pectin) with different concentrations of tannic acid were prepared by a self-assembly method [35]. In brief, 1 mL of modified pectin (500 µg/1 mL) was mixed with 250, 500, 1000, 1500, and 2500 µg (0.25, 0.5, 1.0, 1.5, 2.5 mg) of tannic acid in 10 mL glass vials with a magnetic bead. These solutions were made with MilliQ water. These solutions were exposed to tip sonication using a probe sonicator (200 W, VirSonic Ultrasonic Cell Disrupter 100, The VirTis Company, Woburn, MA, USA) for 2 min. This step promoted the uniformity of molecules in dispersions to favor self-assembly. These solutions were stirred overnight by a magnetic stirrer at a speed of 400 rpm (MS-H-S10, Scilogex, Rocky Hill, CT, USA). The visible aggregates that were stuck to the magnetic bead (usually very minute quantity) were removed from self-assembled pectin-tannic acid nanocomplex (PT-NCs) or modified pectin-tannic acid complex (MPT-NCs) solutions using centrifugation at 1450 relative centrifugal force (rcf) (Sorvall ST 8 Centrifuge, Thermo Fisher Scientific, Suzhou, China). This occurred only due to excessive cross-linking of pectin(s) with higher amounts of tannic acid due to poor stability. This was often observed with pectin A and pectin C. Modified pectin was capable of achieving uniform nanocomplex solutions with a very minute to negligible quantity of aggregates. The PT-NC and MPT-NC solutions were individually purified using dialysis (2K cut off, Millipore Sigma, St. Louis, MO, USA) to remove free TA and pectin. A final and uniform MPT-NC nanocomplex ratio was selected based on a smaller particle size and distribution using DLS measurements.

A stable ratio of modified pectin:tannic acid (1:1, 0.5 mg:0.5 mg) was confirmed to be optimal with a smaller size and highly dispersed in aqueous medium. This ratio of modified pectin tannic acid nanocomplex (MPT-NC) was used throughout our studies. To prepare dye or drug loaded MPT-NCs, a simple solvent evaporation method was employed [36]. A detailed protocol was presented in our earlier publications [35,37,38]. In this method, the solutions of 150 µg 6-coumarin (in 150 µL acetone), or 150 µg drug (gemcitabine, 5-fluorouracil or irinotecan, in 150 µL acetone), or introduced in 0.5 mg of modified pectin (500 µg/1 mL) were mixed with 0.5 mg tannic acid. The total volume was 1 mL. During this loading process, tannic acid could assemble anticancer drugs through hydrogen bonding, as demonstrated earlier [36,38]. Overnight stirring allows acetone to evaporate, which is used to dissolve the drug/dye. The chosen formulation was very stable, but un-entrapped drug was precipitated along with pectin, which was separated by centrifugation at 1450 rcf. The separated aggregates containing coumarin-6 dye or gemcitabine, 5-fluorouracil, and irinotecan were extracted using an acetonitrile:ethanol mixture over a day at room temperature. These solutions were filtered using 0.45 µm filters to estimate drugs that were un-trapped. The un-trapped/un-encapsulated drugs were estimated using the HPLC method employing our published protocols [35,38,39,40,41]. Encapsulation amounts were calculated from the total amount of dye/drug used for generating MPT-NCs and subtracting the dye/drug amount. This was then converted into loading efficiency. The drug loaded modified pectin tannic acid nanocomplexes were called drug MPT-NCs. This process was repeated until we achieved a desired loading efficiency, i.e., > 92–95% of introduced drug in the nanoformulation.

### 2.4. Particle Size Measurement

The particle size and distribution of pectin-tannic acid-NCs and modified pectin-tannic acid-NCs were determined using the dynamic light scattering (DLS) principle [36]. The z-average volume-based diameter counts of nanocomplexes were measured on a Zetasizer (Nano ZS, Malvern Instruments, Malvern, UK) at 25 °C. For these measurements, the nanocomplex suspensions were diluted with water in a ratio of 1:10 and briefly sonicated for 30 s using a probe sonicator (VirSonic Ultrasonic Cell Disrupter 100, The VirTis Company, Gardiner, NY, USA). Our studies suggested that a 30 s probe sonication did not alter the particle fate or drug leaching. Additionally, the sonication step was performed on an ice batch, which protected particle integrity. The sonication step allowed particle to be suspended uniformly, which would not interfere much with particle counts or sample quality when measuring on an instrument. The particle size data were reported as a cumulative average of 3 min readings. Particle size analysis was also carried out under different parameters such as time, temperature, pH, and salinity. The zeta potential of these nanocomplexes were evaluated using the laser Doppler velocimetry method with a Zetasizer. For these measurements, nanocomplexes were dispersed in 1× PBS, and zeta potential counts were acquired for three measurements of 20 runs (90 s) with a gap of 120 s equilibrium. Zeta potential data were documented as the average of three measurements.

### 2.5. Transmission Electron Microscopy 

TEM analysis was carried out to determine the shape and morphology of dry state PT-NCs and MPT-NCs [36]. This was performed with the help of the JEOL 200EX transmission electron microscope (JEOL Ltd., Tokyo, Japan). The aqueous nanocomplex solution (2–3 drops) was placed on a permeable carbon coated copper grid (150 mesh, Electron Microscopy Sciences, Hatfield, PA, USA), and excess nanocomplex solution was removed by a piece of fine filter paper. Then, nanocomplexes were stained with uranyl acetate for better contrast imaging. The preparation of the sample was achieved by water evaporation under a chemical hood. Since pectin shrinks after water evaporation unlike other polymer nanoparticles, its morphology was distinct. Images of nanocomplexes were captured at a magnification of 50,000× and operating at 80 kV.

### 2.6. Scanning Electron Microscopy

SEM morphological evaluation was performed on a Nova Nano SEM 650 Field Emission System (FEI Inc., Hillsboro, OR, USA). For this study, the MPT-NC formulation was lyophilized using a freeze-dryer (Labconco, Kansas City, MI, USA) and deposited physically on a black tape stick on an SEM stage. The sample on the tape was allowed to dry after leaving them in an vacuum sealed incubator. This step allowed the sample to achieve a uniform deposition on the tape. The sample was gold sputtered before imaging. The sample was viewed using microscopy at 20 kV under a high vacuum with field free lens mode.

### 2.7. Fourier Transform Infrared Spectroscopy

For FTIR analysis, nanocomplex formulations were lyophilized using a freeze-dryer (Labconco, Kansas City, MI, USA) to achieve a dry and puffy form. Other parent compounds were used in their solid dry powder form. The FTIR spectra of nanocomplexes were measured by placing on a Diamond/ZnSe Attenuated Total Reflection crystal plate [36]. Spectral data were acquired in the range from 4000 to 650 cm^−1^ with a resolution of 4 cm^−1^ using a PerkinElmer Spectrum 100 FTIR spectrometer (Waltham, MA, USA). Data presented an average of 36 runs.

### 2.8. Cellular Uptake Study

To evaluate the cellular uptake of nanoparticles, we labeled coumarin-6 in MPT-NCs as described in Section 2.3. Coumarin-6 labeling offered rapid visualization and tracking of MPT-NCs in cells. Therefore, cellular uptake of the coumarin-6 labeled MPT-NC formulation was assessed in HPAF-II and PANC-1 cells using two distinct cellular binding methods, fluorescence microscopy (qualitative) and flow cytometry (quantitative) [35]. For these studies, cells were seeded in a 6 well plate at a density of 50,000 cells per well. These cells were treated with 2.5, 5, and 10 µg containing coumarin-6 labelled MPT-NCs for 3 h (dose dependent study) or 5 µg containing coumarin-6 labelled MP-NCs for various time points of 1, 3, and 6 h. After the indicated time points, cells were washed with 1× PBS twice and supplemented with phenol red free medium for imaging. Green fluorescence containing MPT-NCs in cells was imaged using an EVOS^®^ Fluorescence Imaging System (AMF4300, Life Technologies, Carlsbad, CA, USA) with a 20× water immersion objective. Using the same experimental conditions and treatments, cells were trypsinization, centrifuged, and collected in phenol red free medium for flow cytometry analysis. In this procedure, cells were passed through the FITC channel (fluorescence measurements at λex: 485 nm and λem: 520 nm) in a NovoCyte Flow Cytometer (ACEA NovoCyte^®^ 1000, ACEA Biosciences, Inc., San Diego, CA, USA). Each dataset was a representation of 10,000 cells and mean fluorescence intensity (MFI) counts. The MFI was presented from a triplicate measurement. It is important to note that coumarin-6 is a fluorescent dye in MPT-NCs, which could be leached out from MPT-NCs with time. In order to minimize the leaching factor, we performed experiments only for early time points (less than 6 h). Additionally, leached out dye would be precipitated in cell culture medium, which could be removed via centrifugation [37].

### 2.9. In Vitro Cytotoxicity

The in vitro cytotoxicity evaluation of blank MPT-NCs, free drugs, and drug MPT-NCs against HPAF-II and PANC-1 was assessed by the MTT assay [38]. Briefly, 5000 cells were seeded in a 96 well plate with 200 µL of medium in each well. These cells were allowed to attach to the plate for 24 h. Then, the culture medium was replaced with various concentrations of free drugs or the equivalent drug MPT-NCs for 48 h. Untreated and blank MPT-NCs served as controls. After 48 h of treatment, the culture medium was replaced with fresh medium containing the MTT reagent (5 mg/mL) in each well and incubated for 2 h. Then, the supernatant was removed, and 100 µL DMSO were added in each well. The cell viability was measured in a micro plate reader (Cytation 3, BioTeK) at 490 nm. The experiment was done in triplicate and repeated at least twice. Cell viability was calculated using our previously published procedure [38]. The concentration of drug/drug MPT-NCs to inhibit cell proliferation by 50% (IC_50_) was calculated using nonlinear regression analysis with GraphPad Prism 8 (Free trial version, San Diego, CA, USA).

### 2.10. Clonogenic Formation Assay

The colony formation ability of HPAF-II and PANC-1 cells after treatment with free drug or drug MPT-NCs was performed using a clonogenic forming assay [38]. In this assay, 250 cells were seeded in each well of a 12 well plate. Cells were treated with varied concentrations of drug/drug MPT-NCs after cells were attached to the plate. Plates were set in the incubator for the next 15 days with intermittent media change after every 3 days. After 15 days, cells were fixed using cold methanol for 30 min at room temperature after a 1× PBS wash. To visualize colonies, hematoxylin staining was adopted for 30 min. The visible colonies were counted, and data were presented as an average of triplicates.

### 2.11. Statistical Analysis

All the data were expressed as the mean ± standard error of mean (SEM). The statistical significance among treatments was determined from the one-way ANOVA post-hoc Tukey HSD test [42]. A *p*-value ≤ 0.05 was considered to be significant.

## 3. Results

The aim of this study was to construct a novel nanocomplex formulation based on pectin(s). Pectin is a polysaccharide that has major roles as a stabilizer and pharmaceutical excipient. Pectin can serve as a self-targeting agent to cancer cells via β-galactose units, which can bind specifically to galectin-3 adhesive molecules [43,44]. Thus, pectin-based nanocarriers can be suitable to deliver therapeutic drugs to cancer cells. Considering this aspect, we constructed a pectin-based nanocarrier by cross-linking commercially available pectin (pectin A, pectin C, or modified pectin) with tannic acid, following a simple cross-linking method through a self-assembly process, referred to as PT-NCs or MPT-NCs. The formulation formed as nanocomplexes by hydrogen bonding through a self-assembly process.

### 3.1. Optimization of Pectin-Tannic Acid Nanocomplexes

**Figure 1:**
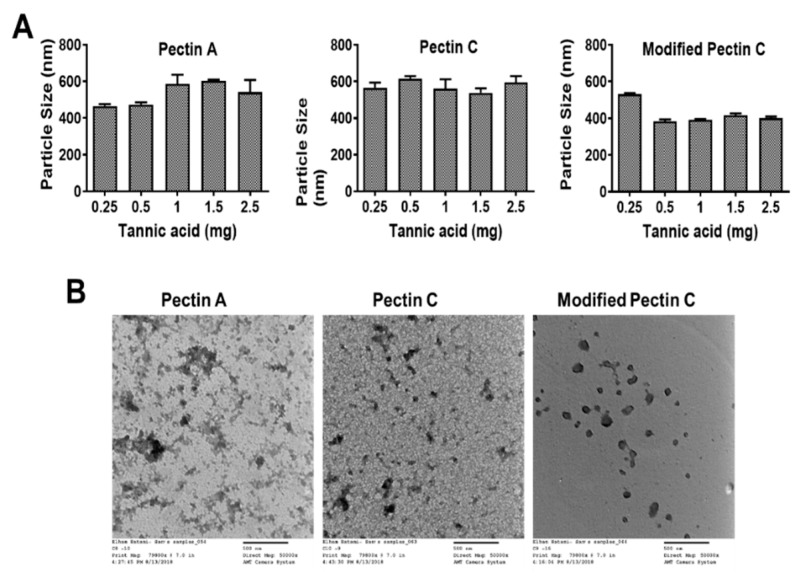
Effect of tannic acid content in the formation of pectin-tannic acid (TA) nanocomplexes. (**A**) Particle size variation upon changing the TA content in the preparation of pectin A-, pectin C-, and modified pectin C-tannic acid nanocomplexes. Formulation composition: 0.5 mg modified pectin, 0.25–2.5 mg tannic acid (the total volume of reaction was 1 mL). The particle size data was reported as a cumulative average of 3 min readings. (**B**) Morphological variation of pectin-tannic acid nanocomplexes. Representative transmission electron microscopic images of pectin A-, pectin C, and modified pectin C-tannic acid nanocomplexes. Direct magnification 50,000×. The scale bar on TEM images represents 500 nm.

The dynamic light scattering particle size distribution of pectin A- (between 455 and 675 nm) and pectin C- (between 497 and 663 nm) showed a higher particle size distribution compared to modified pectin- (between 378 and 541 nm) tannic acid nanocomplexes (Figure 1A). The polydispersity index in all these formulations was observed between 0.12 and 0.26. A higher polydispersity was observed when loose or heterogenous cross-links were formed during the nanocomplexes’ generation. However, such polydispersity was within the range for a stable formulation. Tannic acid (0.5 mg) composed MPT-NC showed the optimal size distribution (383.7 ± 10.74 nm) in aqueous media (Figure 1A). Compared with traditional pectin (pectin from apple and pectin from citrus), the modified pectin from citrus (PectaSol-C) showed a uniform and spherical shape of particle formation and particle size distribution. In the case of pectin A- and pectin C-based tannic acid complexes, they showed smaller or the lowest particle sizes at the lower TA concentrations. However, there was no significant change noticed even after the increase of TA concentration. This indicated that pectin could form cross-links even at the lowest concentrations. In the case of modified pectin C, at the lowest concentration, it may form loosely cross-linked self-assemblies. A further increase of the concentration of TA offered a regulated cross-link network, which showed a lower particle size of MPT-NCs.

The TEM micrograph images of MPT-NCs showed an ideal spherical shape morphology (Figure 1B). The mean diameter of the MPT-NCs was about 80–154 nm (Figure 1B). The difference between DLS and TEM size data was primarily due to the factor of the measurement environment. DLS provided the size of particles that were in suspension, which were always higher due to the hydrodynamic volume of polymers (in this case, pectin) and the swollen stage of tannic acid layers in pectin-tannic acid complexes; whereas TEM size data showed the particles in the dried state where all polymer networks were collapsed.

The overall quality of particle formation in pectin A and pectin C with TA complexes was not clear, but it showed cluster formation when they were dried out on the TEM grid. This may be due to excessive interaction between pectin and tannic acid. While MPT-NCs showed a somewhat clear morphology, they were not like any conventional spherical polymer-based nanoparticles. However, the particles were somewhat spherical in nature due to the lack of excessive stabilizers (such as polyvinyl alcohol, commonly used in PLGA NPs).

**Figure 2:**
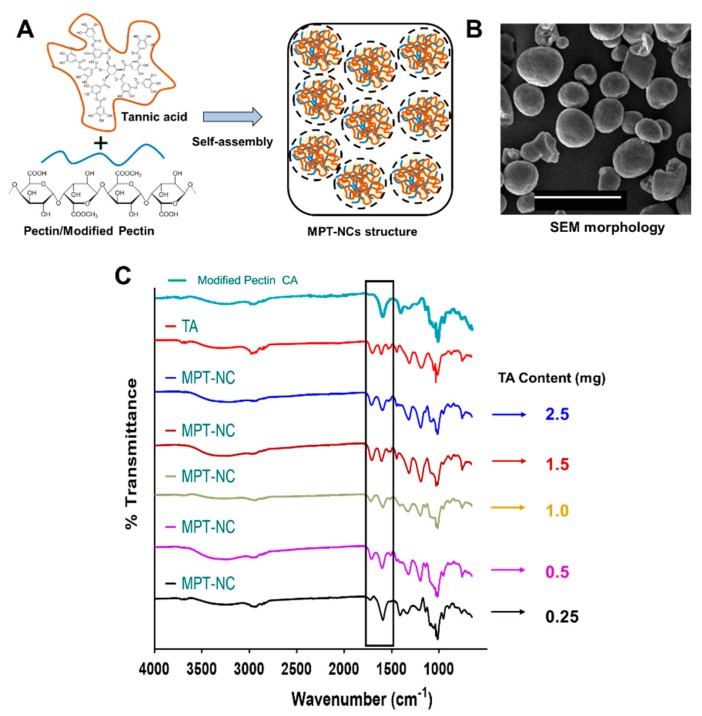
Confirmation of the formation of nanocomplexes based on modified pectin and tannic acid (MPT-NCs). (**A**) Schematic representation of the self-assembly formation process for producing modified pectin-tannic acid nanocomplexes. (**B**) Representative scanning electron microscopy image of MPT-NCs. Direct magnification 10,000×. (**C**) ATR-FTIR spectra of modified pectin, TA, and MPT-NCs. Data present the average of 32 scans. The highlighted portion on FTIR spectra indicates modified pectin and tannic acid cross-linking due to hydrogen bonding between carboxylic and hydroxyl groups.

Considering these data, the self-assembly of MPT-NCs was proposed as shown in Figure 2A. On the other hand, pectin from apple and citrus showed highly aggregated particle structures and no specific morphological conferment. The higher aggregative behavior attributed to these pectin-nanocomplexes may be due to decreased surface tension and increased intra- and inter-molecular aggregation and adhesion. Further, the self-assembly formation was further confirmed through the scanning electron microscopy method (Figure 2B). The SEM image clearly depicted not only a spherical morphology, but a slightly cross-linked or porous nature of these nanocomplexes. These data demonstrated that modified pectin could be a better choice for the construction of nanocarriers for drug delivery application.

### 3.2. Spectral Analysis Confirmed the Presence MPT-NCs’ Formation

FT–IR spectral analysis was used to confirm the presence of modified pectin and tannic acid molecules in MPT-NCs’ formulations. The broad and sharp absorption bands at 3350–3261 cm^−1^, 2930–2928 cm^−1^, 1609–1601 cm^−1^/1409–1402 cm^−1^, and 1027/1098 cm^−1^ were attributed to O–H stretching (galacturonic acid units), C–H vibrations (CH, CH_2_, and CH_3_ stretching), asymmetric and symmetric carboxylate (COO^−^) group stretching, and ether link (C–O–C) stretching for modified pectin (Figure 2C, teal colored spectrum). TA alone showed its characteristic absorption peaks at 3280 cm^−1^, 1725/1697 cm^−1^, and 1187/1016/1066 cm^−1^ due to phenolic O–H stretching, C–O stretching, and C–O–C vibrations (Figure 2C, red color spectrum). In the case of the FT–IR spectrum of MPT-NCs, the formulation exhibited a clear combination of modified pectin and tannic acid related peaks as mentioned above (Figure 2C, black to blue color spectrum). We note that many peaks overlapped due to similar functional groups that were present in both compounds. However, a distinct variation was noticed at 1609–1601 cm^−1^ due to cross-linking through hydrogen bonding, which represents higher amounts of modified pectin (black spectrum). This portion of the peak window is highlighted with a box in the FTIR spectra.

### 3.3. MPT-NCs’ Formulation Is Stable for Therapeutic Application Purposes

**Figure 3:**
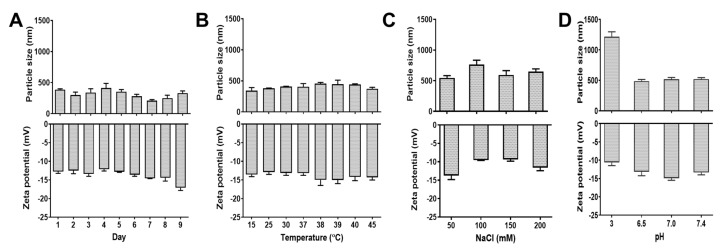
Stability and environmental influence for MPT-NCs’ formulation as a function of time, temperature, pH, and salinity. Dynamic light scattering measurement of particle size and zeta potential of MPT-NCs for (**A**) time (Day 1 to Day 9), (**B**) temperature (15 to 45 °C), (**C**) salinity (25 to 200 mM NaCl), and (**D**) pH (3 to 7.4). The data presented are expressed as the mean ± SEM, *n* = 3. Particle size measurements were the accumulation of 3 min readings, and zeta potential measurements were of 20 measurement for 90 s.

In order to examine the stability of the formulation to extrapolate how particles might distribute over a period, stored MPT-NCs in aqueous media were measured for particle size for a little over a week (nine days). These data shown in Figure 3A showed there was no significant difference in the particles size range for all days. This indicated that MPT-NCs were uniformly distributed in aqueous medium and stable. Such stability was noticed until 30 days (visually observed, no precipitation of particles). Additionally, there was no change observed in their zeta potentials (−12 to −18 mV). This suggested that MPT-NC was a favorable formulation to examine therapeutic applications (in this case, in vitro studies were conducted for over a week). Therefore, this MPT-NC was used for future in vitro experiments.

### 3.4. MPT-NCs Induce Physical Stability

Next, the comparative particle behavior of MPT-NCs was conducted using DLS particle size and zeta potential measurements by varying the environmental conditions, such as temperature, pH, and NaCl concentration. All these DLS results are shown in Figure 3B–D. This nanocomplex formulation showed a slight increase in particle size with the increase temperature from 15 to 39 °C and, after that, lowered at 40 °C (Figure 3B). This may be attributed due to tight cross-linking at moderate temperate compared to low temperature. Similarly, a 50 mM saline concentration proved to be perfect media to get a nanocomplex with tight networks, thus showing a lower particle size range (Figure 3C); whereas a further increase of saline concentration did not result in reducing the intra- and inter-molecular particle interaction. Therefore, a higher particle size of the formulation was attained. In the case of lower pH 3, the particle size of MPT-NCs increased to a greater extent (over 1220 nm), but other pH values (6 to 7.4) showed a particle size less than 465 nm (Figure 3D). Additionally, the zeta potential of MPT-NCs in all three distinct changes in the environment (temperature, pH, and salinity) did not change significantly. All data lied in between −18.4 and −9.68 mV. Altogether, these results indicated no significant change in the formulation’s physical characteristics over multiple factors. This could be attributed to the fact that modified pectin and tannic acid led to tight intermolecular cross-links within a single nanocomplex particle. Further, the cross-linked networks in the particles did not dissociate or flocculate from these integral nanocomplex structures.

### 3.5. MPT-NCs Facilitate Internalization in Cancer Cells

**Figure 4:**
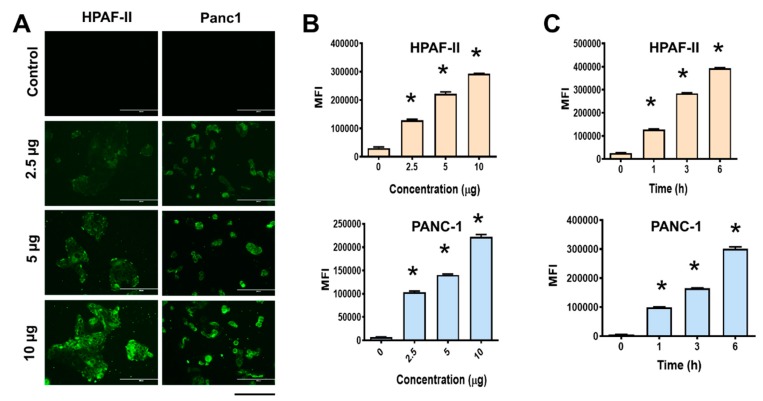
Cellular uptake of MPT-NCs by pancreatic cancer cell lines. Coumarin-6 dye was conjugated with MPT-NCs to image and track nanoparticles under a microscope and by flow cytometry. (**A**,**B**) Dose dependent cellular uptake behavior of MPT-NCs in HPAF-II and PANC-1 cells. (**A**) Fluorescence images and (**B**) flow cytometry-based cellular update phenomenon of 2.5, 5, and 10 µg Coumarin 6-containing MPT-NCs for 3 h of incubation. The scale bar on fluorescence images indicates 200 µm. (**C**) Flow cytometry cellular uptake pattern of 5 µg Coumarin 6-containing MPT-NCs at 1, 3, and 6 h, respectively. Data are expressed as the mean ± SEM, *n* = 3. * *p* < 0.05 of MPT-NCs treatment vs. control cells.

The HPAF-II and PANC-1 cells were used to examine the cellular uptake and distribution of MPT-NCs. For visualization purposes, a green dye 6-coumarin was tagged. The detection of MPT-NCs was done using fluorescence microscopy and a flow cytometer. From the fluorescence microscopy results, as shown in Figure 4A, both cell lines exhibited that with the concentration increasing from 2.5 to 10 µg of dye equivalent MPT-NCs, the accumulation of MPT-NCs was also increased. A similar phenomenon was noticed in the case of time dependent increased uptake of MPT-NCs at a constant time point. Besides this, the accumulation of dye MPT-NCs was used to monitor the extent of uptake in cancer cells by flow cytometer through green fluorescent dye levels in the fluorescence (FL1) channel. These data presented that higher mean fluorescence intensity levels appeared at higher time points and concentrations, which complemented the microscopic uptake pattern data (Figure 4B,C). Overall, these data suggested that MPT-NCs were capable of penetrating inside the cancer cells and carrying drug (dye) molecules in a time and dose dependent fashion.

### 3.6. MPT-Nanocomplexes Improve the Therapeutic Effects of Anticancer Drugs

**Figure 5:**
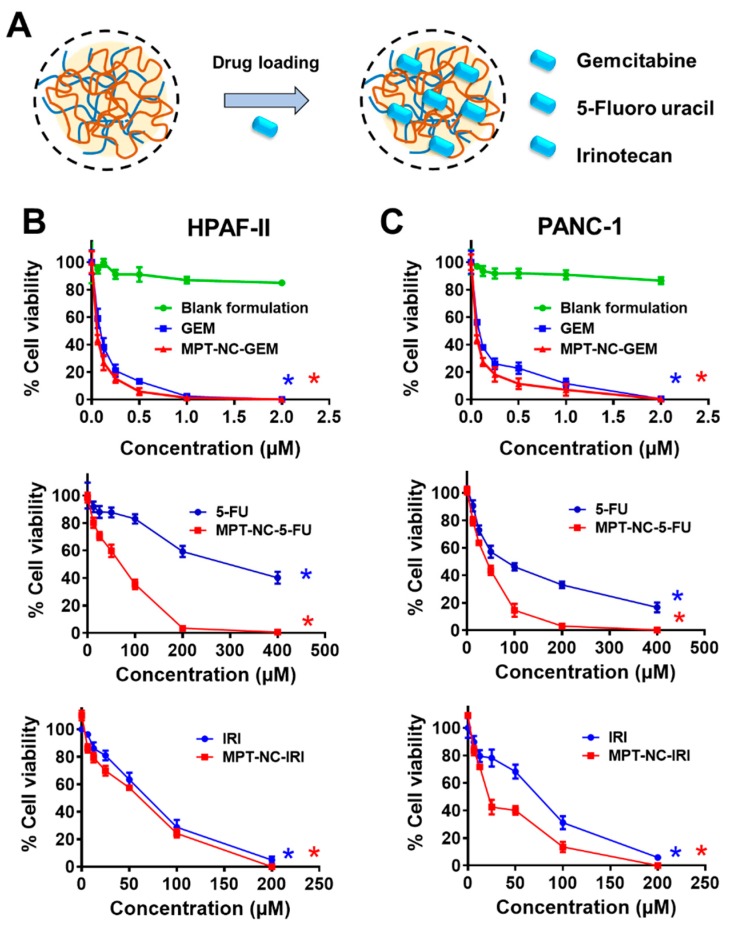
Anti-cancer potential of MPT-NCs against pancreatic cancer cell lines. (**A**) Schematic illustration of drug loading procedure in MPT-NCs. (**B**,**C**) Cell viability of HPAF-II and PANC-1 as a function of GEM, 5-FU, and IRI concentration and drug containing MPT-NCs. * *p* < 0.05 of GEM, 5-FU, and IRI concentration and drug containing MPT-NCs compared to un-treated/control cells. Data are presented as the mean ± SD, *n* = 5.

The primary role of the nanoformulations was associated with their extent of uptake and accumulation throughout the cells and their consequent release of loaded therapeutics. The entire preparative drug encapsulation in modified pectin nanocomplex approach is presented in Figure 5A. The drug release in its active form was reasonable for inducing anticancer potential. To determine the anticancer effects of drug containing MPT-NCs, the MTT cell viability assay was employed. These effects were compared with free drugs (5-FU, GEM, and IRI) in solution. The cell viability of HPAF-II and PANC-1 cells treated with GEM and 5-FU containing MPT-NCs was slightly lower than those treated with free GEM and 5-FU drug solutions for 48 h (Figure 5B,C). The calculated IC_50_ values of GEM solution and GEM MPT-NCs were 107.5 and 70.74 nM (HPAF-II) and 116.5 and 71.92 nM (PANC-1), respectively. Similarly, 5-FU solution and 5-FU MPT-NCs exhibited IC_50_ values > 150 and 88.8 µM (HPAF-II) and 121 and 44.9 µM (PANC-1), respectively. A similar trend was observed for IC_50_ values of IRI and IRI MPT-NCs: 81.15 and 50.41 µM (HPAF-II) and 97.71 and 35 µM (PANC-1), respectively. These data indicated that MPT-NCs efficiently delivered drugs to cancer cells in their active form.

**Figure 6:**
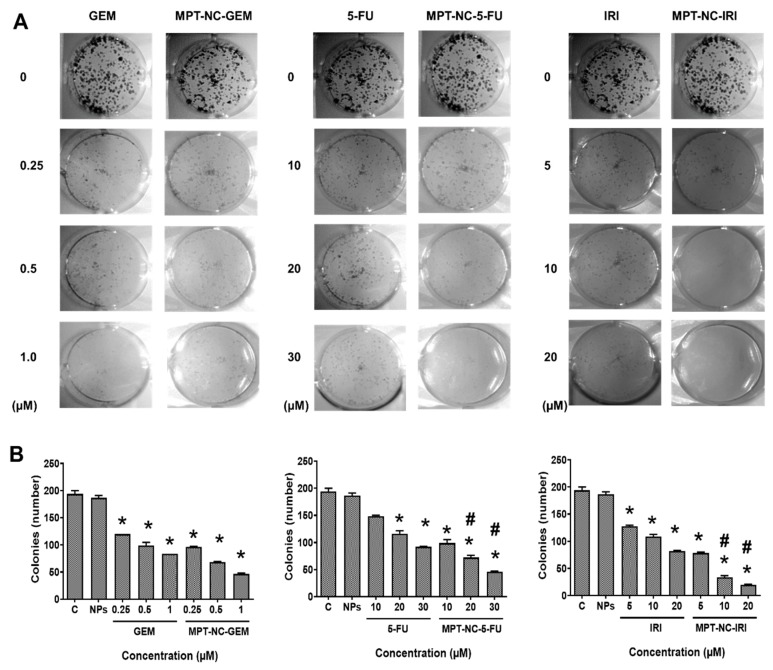
MPT-NCs inhibit the colony formation of PANC-1 cells. (**A**) Representative images of colonies for the predetermined treatment groups (control/blank MPT-NCs and drug containing MPT-NCs). (**B**) Total number of colonies quantified using ChemDoc software. Data represented as the mean ± SEM, *n* = 3. * *p* < 0.05 of GEM, 5-FU, and IRI concentration and drug containing MPT-NCs compared to un-treated/control cells, and # *p* < 0.05 of drug containing MPT-NCs compared to GEM, 5-FU, and IRI treated cells.

To examine whether the drug MPT-NCs induced long-term anticancer potentials, we performed the colony formation assay (Figure 6). Blank MPT-NCs without drug(s) showed a very similar number and size of colonies as the control group (no treatment group) (Figure 6A). Free drug solutions showed a dose-dependent decrease of colonies (Figure 6A). At higher concentrations, the size of colonies became very small, and less than 10% of colonies were observed; whereas drug MPT-NC treatment followed a similar trend as the free drug group in the diminishing size and number of colonies (Figure 6A). However, at a higher concentration of drug, MPT-NCs showed almost no colonies of cancer cells. The quantified data of the colony formation assay are presented in Figure 6B. These results indicated that blank MPT-NCs were not toxic, but they could potentiate drug effects by efficiently delivering drugs into cancer cells.

Altogether, modified pectin-tannic acid nanocomplexes were safe drug delivery vehicles and helped to increase the anticancer activity of parent drugs, 5-fluorouracil, gemcitabine, and irinotecan, against pancreatic cancer cells by efficient internalization.

## 4. Discussion

Biodegradable nanoparticle formulations have been widely used as a drug delivery system due to prolonging the biological half-life of the therapeutics, while reducing the systemic side effects. Among many biodegradable natural polysaccharides, pectin, primarily composed of poly-d-galacturonic acid bonded via α-1,4-glycosidic linkage, is being largely investigated as a colloidal stabilizer, gelling, thickening, and preventive/therapeutic agent [14,45]. Pectin molecules can act as a source for the suppression of cancer progression [46,47,48]. Furthermore, this molecule offers several advantages due to active molecules, which include easy complexation and self-assembly of small or larger molecules [14]. Pectin as a polymer emulsifier can influence various physico-chemical characteristics including the size and distribution of the formulation, the morphology of particles, the zeta potential, and the drug loading and release characteristics. However, alone, pectin cannot serve as good colloid system due to its inherent aggregation characteristic. Thus, the complexation of pectin with other small/biological molecules through various electrostatic interactions such as hydrogen bonding, ion dipole forces, and hydrophobic interactions can result in a nanocomplex formulation. This way, nanocomplexes can be crafted by altering the chemical composition and stimuli factors. Such pectin-based therapeutic formations may offer highly homogeneous, safe, and non-toxic therapeutics with improved therapeutic benefit.

Many nanocarriers commonly require a large amount of surfactant to achieve a stable nanoparticle formulation. In this work, we employed tannic acid as a cross-linker for modified pectin to achieve a well-dispersed nanocomplex formulation. This study reported the feasibility of the nanocomplex formation for MPT-NCs over PT-NCs through DLS, TEM, and SEM analysis (Figure 1, Figure 2 and Figure 3). The amount of tannic acid used in the generation of MPT-NCs had a role in controlling the particle size and distribution. MPT-NCs proved to affect moderately its formulation’s particle size range when applying external stimuli such as temperature, pH, and salinity (Figure 3). More importantly, the MPT-NC formulation was stable over a week of storage without noticing any aggregated fragments in the particle suspensions, as well as in its particle size measurements. Additionally, the visual stability followed for a week indicated the absence of larger aggregated clusters in the solution.

Tannic acid in MPT-NCs also acted as a bridge molecule that could hold both hydrophilic (GEM) or hydrophobic molecules (5-FU/IRI) through self-assembly. Our previous reports indicated that tannic acid was a better hydrophobic anticancer molecule such as paclitaxel and docetaxel compared to many other commonly used pharmaceutical excipients and polymers [36,38]. This study suggested up to ~9.1 to 9.4 wt % of drug could be encapsulated using MPT-NCs for gemcitabine, 5-FU, and irinotecan when used in a 1 (drug):10 (MPT-NC) ratio. The advised loading efficiency was over 91%. Further investigations are underway to examine improving the loading amount in the MPT-NCs.

The key role of the nanocarrier in cancer therapy is to deliver therapeutics to cancer cells more efficiently. A primary indication of this property could be confirmed through its cellular interaction. Our study demonstrated that the newly generated MPT-NCs could bind and internalize in PanCa cells (Figure 4). We utilized a green fluorescent dye to track MPT-NCs in cancer cells visually and by flow cytometry methods. MPT-NCs were established as responsive to both time and concentration, leading to fast internalization, which indicated their utility in cancer treatments.

Chemotherapy has always been a backbone and the most dependable approach in cancer treatments. Chemotherapy primarily enhances the meaningful survival life-time. Many chemotherapeutic agents have restored the therapeutic benefits for pancreatic cancer patients. Therefore, drug delivery systems with self-targeting capability are always a prime strategy to improve the therapeutic outcome. Various polysaccharide-based nanosystems have proven to be suitable efficient carriers for a few chemotherapy agents. Pectins are considered as tumor targeting agents via β-galactose units, which can bind specifically to the galectin-3 adhesive molecule (overexpressed in various types of cancers) [15,26]. Galectin-3, a β-galactoside-binding protein, is known to be involved in multiple roles in cancer development and progression including tumor cell growth, adhesion, angiogenesis, invasion, anti-apoptosis, and metastasis [49,50,51,52]. It also expresses on other normal organs at lower levels, while overexpressed in fibrosis and inflammation conditions. Therefore, developing a formulation for cancer therapeutics to target galectin-3 for intra-tumoral purposes is ideal. However, in the absence of other disease conditions, galectin targeting may be developed as intra-venous formulations.

Our pectin-tannic acid nanocomplex-based drug formulations demonstrated synergic effects against cancer cells over free drugs (Figure 5 and Figure 6). This was anticipated due to an extended targeted capability of pectin. Additionally, we anticipate animal studies will further elucidate its superior therapeutic applicability. Overall, the results demonstrated the potential benefits of this unique nanosystem for pancreatic cancer treatment.

## 5. Conclusions

In this study, we engineered a novel pectin-based nanocomplex drug delivery system for the treatment of pancreatic cancer cells. Tannic acid was introduced to produce self-assembled cross-linking with pectin. The interface of pectin and tannic acid layers offered an efficient depot for hydrophilic and hydrophobic drugs. This facile approach did not use toxic solvents or surfactants, but contributed a nanocomplex formulation with a stable particle size and morphology. Cellular uptake studies demonstrated a time and dose dependent internalization for improved therapeutic benefit. Cell viability and clonogenic formation assays clearly validated the superior anticancer effects against pancreatic cancer cells. This study reported a promising new nanotechnology strategy for efficient pancreatic cancer therapy.
